# Early predictors of behavioural problems in pre-schoolers – a longitudinal study of constitutional and environmental main and interaction effects

**DOI:** 10.1186/s12887-016-0614-x

**Published:** 2016-06-07

**Authors:** Sara Agnafors, Gunilla Sydsjö, Erika Comasco, Marie Bladh, Lars Oreland, Carl Göran Svedin

**Affiliations:** Division of Child and Adolescent Psychiatry, Department of Clinical and Experimental Medicine, Faculty of Health Sciences, Linköping University, SE-581 85 Linköping, Sweden; Division of Obstetrics and Gynaecology, Department of Clinical and Experimental Medicine, Faculty of Health Sciences, Linköping University, SE-581 85 Linköping, Sweden; Department of Neuroscience, Uppsala University, SE-751 24 Uppsala, Sweden

**Keywords:** Behaviour, Gene-by-environment, Longitudinal, SESBiC-study, Socio-environment

## Abstract

**Background:**

The early environment is important for child development and wellbeing. Gene-by-environment studies investigating the impact of the serotonin transporter gene-linked polymorphic region (*5-*HTTLPR) and the Brain Derived Neurotrophic Factor (*BDNF*) Val66Met polymorphisms by life events on mental health and behaviour problems have been inconclusive. Methodological differences regarding sample sizes, study population, definitions of adversities and measures of mental health problems obstacle their comparability. Furthermore, very few studies included children. The aim of this study was to examine the associations between a broad range of risk factors covering pregnancy and birth, genetic polymorphism, experience of multiple life events and psychosocial environment, and child behaviour at age 3, using a comparably large, representative, population-based sample.

**Methods:**

A total of 1,106 children, and their mothers, were followed from pregnancy to age 3. Information on pregnancy and birth-related factors was retrieved from the Medical Birth Register. Questionnaires on depressive symptoms, child behaviour and child experiences of life events were filled in by the mothers. Child saliva samples were used for genotyping the *5-*HTTLPR and *BDNF* Val66Met polymorphisms. Multiple logistic regression was used to investigate the association between psychological scales and genetic polymorphisms.

**Results:**

Symptoms of postpartum depression increased the risk of both internalizing and externalizing problems. Experience of multiple life events was also a predictor of behavioural problems across the scales. No gene-by-environment or gene-by-gene-by-environment interactions were found. Children of immigrants had an increased risk of internalizing problems and parental unemployment was significantly associated with both internalizing and externalizing type of problems.

**Conclusion:**

This study shows the importance of the psychosocial environment for psychosocial health in preschool children, and adds to the literature of null-findings of gene-by-environment effects of *5*-HTTLPR and *BDNF* in children.

**Electronic supplementary material:**

The online version of this article (doi:10.1186/s12887-016-0614-x) contains supplementary material, which is available to authorized users.

## Background

Behavioural problems in children are risk factors for later adaption and mental health problems [1, 2]. Early identification of risk factors and families at risk of dysfunctional development is thus of great importance for the provision of preventive actions avoiding long-term, negative consequences in childhood and adolescence.

It is plausible to consider the early environment as important for later adaption and wellbeing of the child. Brain development starts already during foetal life and continues during infancy and childhood. Perinatal factors have been studied in relation to mental health in childhood with divergent results [3, 4]. There is however considerable support for an increased prevalence of externalizing problems in preterm children [5]. Moreover, postpartum depression has been shown to exert a risk factor for child behavioural problems in numerous studies [6, 7].

Although the importance of genetic variability on behaviour is generally accepted, studies on genetic factors known to affect mental health in adults and adolescents are sparser when it comes to young children [8–10]. The serotonin transporter gene-linked polymorphic region (*5*-HTTLPR) and the Brain Derived Neurotrophic Factor (*BDNF*) single nucleotide polymorphism Val66Met, are two of the most well-studied polymorphisms for depressive symptomatology and internalizing problems [11]. The serotonin transporter (5-HTT) is regulating serotonergic signalling, and the short (s) allele of the *5-*HTTLPR polymorphism has been associated with less effective 5-HTT expression and availability compared to homozygosity for the long (l) allele [12]. *BDNF* is involved in reparation, plasticity and neurogenesis in the brain, and a single nucleotide polymorphism (SNP) G/A (Val66Met) in the *BDNF* gene has been shown to affect levels of BDNF in the brain. BDNF has previously been shown to act in synergy with serotonin [13].

Exposure to traumatic life events has been shown not only to exert an immediate impact on behavioural problems in young children, but also to affect mental health in the long run [14]. Moreover, it is well known that genetic and environmental factors contribute to the aetiology of psychiatric disorders. Candidate gene-by-environment studies (cGxE), which have literally prospered during the last decades, have demonstrated an interaction effect of childhood adversity and genetic variability [15]. Discordant results of gene-by environment effects related to both *5-*HTTLPR and *BDNF* Val66Met polymorphisms have been found [16–18]. As stated by Duncan and Keller [19] there is a call for well powered direct replication studies. Moreover, given the complexity of psychiatric disorders it is plausible to consider the involvement of multiple genes in the development of behavioural problems. Synergistic effects between the BDNF and serotonin systems on regulation of development and plasticity of neural circuits have indeed been shown, and the interplay between the *5*-HTTLPR and *BDNF* Val66Met polymorphism is likely to have an impact on affective behaviour [13].

In 2006, Kaufman and colleagues presented a significant three-way interaction between the *5-*HTTLPR, *BDNF* and childhood maltreatment [9]. A handful of replication attempts have been made, some successfully replicating the results [20–23] whereas others not [24–26]. However the interpretation and generalizability of the results are limited due to a number of reasons. Firstly, sample characteristics differed markedly between the studies. Two of the samples consisted mainly of university students [24, 25] and the representativeness of the population could thereby be questioned. Other studies were limited by a relatively small number of participants [9, 20], and one study included only females [23]. Secondly, measures and definitions of stress varied across the studies. Self-reports on childhood trauma were used in several studies [20, 22, 23, 25], increasing the risk for recall bias. Thirdly, outcome variables differed between the studies, some using clinical diagnoses of depression [20], or structured interviews for lifetime depression [24] whereas self-report questionnaires were used in the majority of studies. Most studies controlled for sex and if relevant, age and ethnicity, but very few [26] added other variables in the analysis. Behaviour and mental health problems are multifactorial, and the addition of relevant factors can give a more complete picture of depressive symptoms or behaviour problems. Moreover, except from the previously published results from the South East Sweden Birth Cohort (SESBiC) study [27], the original study by Kaufman et al. was the only one conducted on children (age 5–15).

Serotonin and BDNF are involved as neurotrophic factors during neurodevelopment. Thus the study of gene-by-environment interaction in early childhood, including the *5*-HTTLPR and *BDNF* Val66Met could shed light on early moderation by the *5-*HTTLPR and *BDNF* and its consequences for child development and behaviour [28]. A study on early onset depression in 3–5 year old children found an interaction effect of *5*-HTTLPR and stressful life events, such that children homozygous for the short allele were at increased risk for depression in the presence of stressful life events [29]. Other studies also indicate that gene-by-environment interaction effects of both *BDNF* Val66Met and *5*-HTTLPR on emotional development can be seen early in life [30, 31]. Concluding, given the inconsistency of the results from *5-*HTTLPR and *BDNF* gene-by-environment studies on behavioural and psychiatric symptoms, replication studies are more needed than explorations of novel gene-by-environment interaction effects [19].

The primary aim of this study was to explore the importance of the early environment and constitutional factors for child behaviour at preschool age. In particular we aimed to test the gene-by-environment interaction of *5*-HTTLPR, *BDNF* Val66Met and experience of multiple life events on internalizing and externalizing problems at age 3 and to examine potential predictive associations between a broad range of risk factors covering pregnancy and birth, experience of multiple life events and psychosocial environment and child behaviour at age 3. The present study aims to add knowledge to the existing literature by 1) using a comparably large, representative, population based sample, 2) investigating gene-by-gene-by-environment interaction effect on behaviour problems in early childhood which has not previously been studied, and 3) using a virtually similar setup regarding phenotype, genetic variables, environmental moderator and statistical methods compared to previous studies, making it possible to draw conclusions and generalizing the results.

## Methods

### Subjects

This study is part of the South East Sweden Birth Cohort study (SESBiC-study), which began in 1995 with the purpose of early identification of psychosocially burdened families where children were at risk of dysfunctional development. Follow-ups have been carried out at ages 3, 5.5 and 12 and have been reported previously [27, 32–35].

In the SESBiC-study all the mothers of children from a birth cohort born between May 1st 1995 and December 31st 1996 in southern Sweden were asked to take part in the study and 1,723 mothers (88 %) agreed to participate. The mean age of the mothers was 28.2 ± 4.6 years at child birth. Ninety six percent of the mothers (*n* = 1574) were cohabitating, 3.5 % (*n* = 57) were single parents, while 0.5 % (*n* = 8) reported other family arrangements. Most mothers were born in Sweden (*n* = 1 482, 88.6 %), but 6.2 % (*n* = 103) were born in Europe (except Sweden), and 5.3 % (*n* = 88) outside Europe. Of the new-born children, 52.8 % were boys and there were 27 pairs of twins.

### Procedure

The baseline study was carried out at Child Welfare Centres, (CWC) in connection with the routine 3-month check-up. Information about participation was given by the CWC staff. Questionnaires were administered and the mothers were also interviewed by a psychologist.

The 3-year follow-up was carried out in connection with the routine examination of 3-year olds at the CWC. Mothers were asked to fill in questionnaires and medical information was retrieved from the child’s medical records. One child was deceased and 1,452 (84 %) agreed to participate.

The genetic sample was collected at the 12-year follow-up. An information letter and a consent form were sent to parents (i.e. legal guardians) and had to be signed before participation in the study. The children were asked to provide saliva samples and fill out questionnaires as part of a larger study. Two children were deceased, ten had moved out of the country and 24 were learning disabled and could not therefore participate, which left 1,687 eligible participants of whom 1,106 provided saliva samples suitable for DNA analysis.

### Instruments

The Edinburgh Postnatal Depression Scale (EPDS) [36] is a widely used 10 item self-report questionnaire designed to screen for postnatal depression. EPDS refers to the 7 days preceding completion of the form and was filled out by the mothers at baseline.

The Life Stress Score (LSS) is a 50-item semi-structured interview form, consisting of three main domains regarding social, medical and psychological conditions related to the mother. The LSS has been used previously in a Swedish population-based study [37] and was filled out by a psychologist after interviewing the mothers at baseline.

The Child Behaviour Check List/2-3 (CBCL) [38] is a well-known 100 item form assessing child behaviour, focusing on subscales of internalizing and externalizing behaviour respectively. The CBCL was filled out by the mothers at the 3-year follow-up.

A modified 33-item version of the Coddington’s Life Event Scale (CLES) [39] was used to screen for exposure to different life events. The life event form holds virtually the same events as the CLES but evaluates only the occurrence of an event, not the time aspect. The life event form was filled out by the mothers at the 3-year follow-up.

### Medical data

The Medical Birth Register holds medical information on approximately 98–99 % of all births in Sweden. Routine checks are performed by the Swedish National Board of Health and Welfare, concluding that the majority of the variables in the MBR are fairly reliable [40]. From the MBR, information on ante and perinatal birth characteristics was retrieved. The birth characteristics from the MBR were defined as follows: *Small for gestational age (SGA)* was defined as birthweight < -2 SD of the mean weight for the gestational length [41]. *Preterm birth* was defined as being born before gestational week 37, and *Very preterm birth* defined as born before gestational week 32. *Low birth weight* was defined as birthweight below 2500 g, and *Very low birth weight* as below 1500 g. *Apgar score* <7 at five minutes was defined as low in accordance with previous studies [42]. Information on *smoking during pregnancy* was obtained during the first and third trimester. Children of mothers who smoked on one or both of these occasions were compared to children of non-smoking mothers. Infant diagnoses in the MBR were complemented by information on *neonatal illness*, retrieved from the child’s medical record at the 3 year follow-up. Minor injuries during birth, diagnoses regarding small with respect to gestational length, and mild neonatal distress were not considered, whereas infections, hypoglycaemia, anaemia and moderate and severe congenital malformations etc. were included. Children with one or more diagnoses were compared to healthy children. The birth and pregnancy characteristics were finally combined to a *Non-optimal birth characteristics* score, where children with one or more non-optimal birth factors were compared to children with no birth adversity. Permission to access the Medical Birth Register was obtained from the *Regional Ethical Review Board* in Linköping in 2007, and written consent was obtained from parents.

### Genetic analysis

The non-invasive and all-in-one Oragene® DNA Collection Kit (DNA Genotek) was used for the collection, stabilization and transportation of saliva samples. DNA was isolated according to the laboratory protocol for manual purification of DNA. *BDNF* Val66Met A/G SNP (rs6265) and *5*-HTTLPR genotyping was carried out as previously described [43]. The genotyping call was blind to psychosocial data. In order to estimate the quality-rate of genotyping errors, a random repetition of ~13 % of the sample was carried out; the comparison indicated no inconsistencies. The genotypes were in Hardy-Weinberg equilibrium (*5*-HTTLPR (*χ*^*2*^ = 2.73; *p* = 0.10); females (*χ*^*2*^ = 0.44; *p* = 0.51); males (*χ*^*2*^ = 2.67; *p* = 0.10), *BDNF* Val66Met (*χ*^*2*^ = 0.08; *p* = 0.77); females (*χ*^*2*^ = 1.20; *p* = 0.27); males (*χ*^*2*^ = 1.98; *p* = 0.16)). Based on previous findings, we hypothesized that the s allele of the *5*-HTTLPR and the Met allele of the *BDNF* Val66Met would be associated with higher levels of behavioural problems in the presence of experience of multiple life events. Genotypes were analysed separately, not creating dichotomous groups for risk alleles. Thus, in bivariate and regression analyses, the l/l and Val/Val genotypes were used as reference levels, to which the other genotypes were compared.

### Data analysis

A cut-off of 10 was used for the EPDS as used previously for screening purposes [44]. On the LSS, Life Event and CBCL scales, the 90th percentile was set as a cut-off. Pearson correlation was used to test for gene-by-environment correlations, and correlations between independent and dependent variables. Bivariate analyses between genetic markers (*5*-HTTLPR and *BDNF* Val66Met), psychological scale (EPDS), medical data (MBR), socio-demographic variables (LSS and parental unemployment) and Life Events as independent variables and CBCL subscales as dependent variables were performed using logistic regression. Each CBCL subscale was modelled separately. Multivariate analyses, with CBCL scales as dependent variables and other scales as independent variables, were also performed. Parental immigration status (both parents born in Sweden, compared to one or both parents born abroad) and sex of the child were also controlled for. The multivariate analysis consisted of conditional stepwise logistic regression considering full factorial models. Due to the procedure which allows for models containing interactions without corresponding main effects, the models have been corrected for this and further evaluated and reduced to include models with significant main effects and appropriate corresponding interactions, or significant interactions and the corresponding main effects even though the main effects may not be statistically significant. The numbers of models tested for each subscale were: Internalizing 6 models, Anxious depressed 6 models, Withdrawn 6 models, Externalizing 10 models, Aggressive 12 models, Destructive 6 models. Data was also tested for population stratification where *χ*^2^-analysis revealed no significant differences in genotype distribution between children of immigrants and children of non-immigrants. Results are presented with corresponding Odds Ratios (OR) and 95 % Confidence Intervals (CI). A p-value < 0.05 was considered statistically significant. All statistical analyses were performed using IBM SPSS version 22 (IBM Corporation, Armonk, NY).

### Drop-out rate analysis

The total dropout rate was 34.5 % (*n* = 581). There was a difference when comparing immigrant status between participants and non-participants, where 66.3 % (*n* = 987) of mothers born in Sweden (*n* = 1489) took part compared to 55.9 % (*n* = 57) born in Europe (*n* = 102) and 46.0 % (*n* = 40) born outside Europe (*n* = 87) (*χ*^2^ = 18.435, *p* < 0.001). Likewise, differences were found between participants and non-participants when comparison for maternal life stress at baseline was made. Of the mothers with a high life stress score at baseline (*n* = 144), 54.9 % (*n* = 79) took part in the follow-up compared to 65.5 % (*n* = 1027) with a low score at baseline (*n* = 1567) (*χ*^2^ = 6.579, *p* = 0.010). No differences were found between participants and non-participants regarding symptoms of postpartum depression.

## Results

### Prevalence

Frequency characteristics of the cohort are found in Table 1.

**Table 1:** Frequency characteristics of the study population

Variable	N (%)	M/SD or Median/Range
Independent Variables		
*BDNF* Val66Met	1106	
Val/Val	722 (65 %)	
Val/Met	345 (31 %)	
Met/Met	39 (4 %)	
*5-*HTTLPR	1015	
l/l	318 (31 %)	
l/s	473 (47 %)	
s/s	224 (22 %)	
Apgar 5 min (Median/Range)	1079	10/0–10
Low <7	12 (1 %)	
Birthweight (Mean/SD)	1081	3554.01/540.80
Low <2500 g	33 (3 %)	
Very low <1500 g	1 (0 %)	
Gestational length (Median/Range)	1086	40/31–43
Preterm <37 weeks	50 (5 %)	
Very preterm <32 weeks	2 (0 %)	
Small for gestational age	1051	
Yes	21 (2 %)	
Smoking during pregnancy	1106	
Yes	172 (16 %)	
Neonatal illness	1106	
Yes	97 (9 %)	
Non-optimal pregnancy/birth	1106	
Yes	287 (26 %)	
EPDS (Median/Range)	1097	4/0–21
≥ 10	121 (11 %)	
Life events (Median/Range)	980	4/0–18
≥ 90th percentile	62 (6 %)	
LSS (Median/Range)	1106	4/0–19
≥ 90th percentile	79 (7 %)	
Parental unemployment	930	
One or both parents unemployed	109 (12 %)	
Sex	1106	
Boys	562 (51 %)	
Parental immigration status	1106	
Children of immigrants	127 (11 %)	
Dependent Variables		
CBCL Internalizing	983	
≥ 90th percentile	83 (8 %)	
CBCL Anxious Depressed	983	
≥ 90th percentile	52 (5 %)	
CBCL Withdrawn	983	
≥ 90th percentile	57 (6 %)	
CBCL Externalizing	983	
≥ 90th percentile	85 (9 %)	
CBCL Aggressive	983	
≥ 90th percentile	75 (8 %)	
CBCL Destructive	983	
≥ 90th percentile	84 (9 %)	

*BDNF* brain derived neurotrophic factor, *5-HTTLPR* serotonin transporter gene-linked polymorphic region, *EPDS* Edinburgh postnatal depression scale, *LSS* life stress score, *CBCL* child behaviour checklist

### Correlations

No gene-by-environment correlations were found. Strong positive correlations were found between birthweight, gestational length and SGA. Moderate to strong positive correlations were also found between the CBCL scales (Additional file 1).

### Bivariate analysis

#### Internalizing problems

In bivariate analysis, children whose mothers reported symptoms of postpartum depression showed an increased risk of internalizing problems. The same applied when internalizing problems was divided into anxious depressed and withdrawn symptoms, where a three-fold increased risk was seen. Likewise, maternal life stress at baseline increased the risk of internalizing and anxious depressive problems, but results were not significant for withdrawn problems. Life events was also a strong predictor of internalizing problems, both for the total scale and for the subscales (Table 2).

**Table 2:** Bivariate logistic regression predicting internalizing problems at age 3

	Odds Ratio	95.0 % CI for OR	*p*-value
Internalizing ≥ 90th percentile			
BDNF Val66Met			
Val/Met compared to Val/Val	1.088	0.674–1.757	0.730
Met/Met compared to Val/Val	0.712	0.166–3.058	0.648
EPDS	3.838	2.254–6.535	<0.001
Life events	3.321	1.716–6.425	<0.001
LSS	2.394	1.199–4.779	0.013
Parental unemployment	2.749	1.566–4.828	<0.001
Parental immigration status	3.084	1.778–5.350	<0.001
Anxious depressed ≥ 90th percentile			
BDNF Val66Met			
Val/Met compared to Val/Val	1.886	1.069–3.326	0.028
Met/Met compared to Val/Val	0.707	0.093–5.370	0.738
EPDS	3.048	1.569–5.920	0.001
Life events	4.076	1.934–8.587	<0.001
LSS	3.270	1.518–7.046	0.002
Parental unemployment	2.610	1.313–5.188	0.006
Parental immigration status	3.835	2.025–7.263	<0.001
Withdrawn ≥ 90th percentile			
BDNF Val66Met			
Val/Met compared to Val/Val	0.776	0.421–1.430	0.416
Met/Met compared to Val/Val	1.536	0.449–5.256	0.494
EPDS	3.212	1.816–6.410	<0.001
Life events	3.704	1.768–7.763	0.001
LSS	2.095	0.910–4.826	0.082
Parental unemployment	2.549	1.316–4.935	0.006
Parental immigration status	3.716	2.003–6.895	<0.001

*CI* confidence interval, *OR* odds ratio, *BDNF* brain derived neurotrophic factor, *EPDS* Edinburgh postnatal depression scale, *LSS* life stress score, *CBCL* child behaviour checklist. Results concerning Apgar score, Small for Gestational Age (SGA), smoking during pregnancy, birthweight, duration of gestation, neonatal illness, non-optimal pregnancy/birth, sex and *5*-HTTLPR were not statistically significant and are not shown

Logistic regression. Dependent variables: CBCL (internalizing, withdrawn and anxious depressed). Independent variables (0 is used as a reference level): EPDS (0 ≤ 9, 1 ≥ 10), Life Events and LSS (0 < 90th percentile, 1 ≥ 90th percentile), Parental unemployment (0 = both parents employed, 1 = one or both parents unemployed), Parental immigration status (0 = both parents born in Sweden, 1 = one or both parents born abroad)

Carriers of the Val/Met genotype of the *BDNF* Val66Met genotype showed an increased risk of problems on the anxious depressive subscale compared to Val/Val carriers (Table 2). No other effects of the *5*-HTTLPR or *BDNF* Val66Met polymorphisms were seen. Likewise, no effect was seen for prenatal and birth-related factors on internalizing problems at age 3.

Children of whom one or both parents were unemployed showed an increased risk for internalizing problems as well as anxious depressed and withdrawn symptoms (Table 2). Children of immigrants had an increased risk of internalizing problems apparent also on both subscales. No sex differences were found for internalizing problems (Table 2).

### Externalizing problems

In bivariate analysis, maternal symptoms of postpartum depression predicted externalizing problems at age 3, both on the total scale and on the aggressive and destructive subscales. Maternal life stress at baseline increased the risk of externalizing and destructive problems, but significance was not reached for aggressive problems. Experience of multiple life events was also strongly associated with externalizing behavioural problems at age 3, evident also on the aggressive and destructive subscales (Table 3).

**Table 3:** Bivariate logistic regression predicting externalizing problems at age 3

	Odds ratio	95.0 % CI for OR	*p*-value
Externalizing ≥ 90th percentile			
Apgar 5 min	2.380	0.506–11.198	0.272
Non-optimal birth characteristics	1.427	0.886–2.298	0.144
EPDS	2.952	1.705–5.113	<0.001
Life events	4.812	2.610–8.873	<0.001
LSS	3.275	1.728–6.205	<0.001
Parental unemployment	3.419	1.989–5.877	<0.001
Sex	1.480	0.941–2.329	0.090
Parental immigration status	1.948	1.070–3.546	0.029
Aggressive ≥ 90th percentile			
Apgar 5 min	1.214	0.153–9.610	0.855
Non-optimal birth characteristics	1.182	0.704–1.986	0.528
EPDS	2.066	1.111–3.841	0.022
Life events	4.170	2.175–7.995	<0.001
LSS	2.075	0.983–4.379	0.056
Parental unemployment	2.981	1.667–5.331	<0.001
Sex	1.037	0.647–1.661	0.881
Parental immigration status	1.167	0.563–2.417	0.678
Destructive ≥ 90th percentile			
Apgar 5 min	6.371	1.826–22.229	0.004
Non-optimal birth characteristics	1.635	1.021–2.618	0.041
EPDS	3.002	1.732–5.204	<0.001
Life events	2.911	1.481–5.724	0.002
LSS	3.325	1.754–6.304	<0.001
Parental unemployment	3.489	2.027–6.005	<0.001
Sex	1.804	1.133–2.872	0.013
Parental immigration status	2.578	1.463–4.543	0.001

*CI* confidence interval, *OR* odds ratio, *EPDS* Edinburgh postnatal depression scale, *LSS* life stress score, *CBCL* child behaviour checklist. Results concerning Small for Gestational Age (SGA), smoking during pregnancy, birthweight, duration of gestation, neonatal illness, non-optimal pregnancy/birth, *BDNF* and *5*-HTTLPR were not statistically significant and are not shown

Logistic regression. Dependent variables: CBCL (externalizing, aggressive and destructive symptoms). Independent variables (0 is used as a reference level): Apgar (0 < 7, 1 ≥ 7), Non-optimal birth characteristics (0 = no birth adversity, 1 = one or more birth adversities), EPDS (0 ≤ 9, 1 ≥ 10), Life Events and LSS (0 < 90th percentile, 1 ≥ 90th percentile), Parental unemployment (0 = both parents employed, 1 = one or both parents unemployed), sex (0 = girls and 1 = boys), Parental immigration status (0 = both parents born in Sweden, 1 = one or both parents born abroad)

Non-optimal birth characteristics and a low Apgar score at birth carried a slight increase of risk of destructive problems, but no other effect was seen for the pregnancy and birth-related variables (Table 3). For the externalizing scales, there were no influences from either *5-*HTTLPR or *BDNF* Val66Met polymorphisms.

Children with one or both parents unemployed had an increased risk for externalizing problems, which was evident also on the subscales aggressive and destructive (Table 3). Boys exhibited significantly more destructive problems than girls, but there were no sex differences for externalizing or aggressive problems. Likewise, parental immigrant status was significantly linked to externalizing and destructive problems, but not related to aggressive problems (Table 3).

The analyses for internalizing and externalizing problems were also run with genetic polymorphisms as dichotomous variables and split by sex, however the results differed only marginally from those presented.

### Multivariate analysis

In multivariate analysis, a few variables predicting internalizing problems were consistent over all three models. Maternal symptoms of postpartum depression more than doubled the risk of internalizing problems. Experience of multiple life events was the strongest predictor of internalizing problems, giving almost four times the risk of anxious depressive problems. Likewise, children of immigrants were at greater risk of internalizing problems compared to children of non-immigrants (Table 4).

**Table 4:** Multiple logistic regression predicting internalizing problems at age 3

	Odds ratio	95.0 % CI for OR	*p*-value
Internalizing ≥ 90th percentile	r^2^ = 0.08		
EPDS	2.932	1.595–5.389	0.001
Life events	2.263	1.029–4.980	0.042
Parental unemployment	2.194	1.207–3.988	0.010
Parental immigration status	2.171	1.151–4.095	0.017
Anxious depressed ≥ 90th percentile	r^2^ = 0.09		
EPDS	2.625	1.317–5.233	0.006
Life events	3.804	1.766–8.194	0.001
Parental immigration status	3.048	1.572–5.908	0.001
Withdrawn ≥ 90th percentile	r^2^ = 0.08		
EPDS	2.749	1.410–5.361	0.003
Life events	3.563	1.663–7.634	0.001
Parental immigration status	2.794	1.451–5.382	0.002

*CI* confidence interval, *OR* odds ratio, *EPDS* Edinburgh postnatal depression scale, *CBCL* child behaviour checklist. Results concerning Apgar score, Small for Gestational Age (SGA), smoking during pregnancy, birthweight, duration of gestation, neonatal illness, non-optimal pregnancy/birth, sex, LSS, *BDNF* and *5*-HTTLPR were not statistically significant and are not shown

Multiple logistic regression. Dependent variables: CBCL (internalizing, withdrawn and anxious depressed). Independent variables (0 is used as a reference level): EPDS (0 ≤ 9, 1 ≥ 10), Life Events and LSS (0 < 90th percentile, 1 ≥ 90th percentile), Parental unemployment (0 = both parents employed, 1 = one or both parents unemployed), Parental immigration status (0 = both parents born in Sweden, 1 = one or both parents born abroad)

In a similar manner as with the internalizing scales, a couple of variables were found to consistently influence externalizing problems. Symptoms of postpartum depression doubled the risk of externalizing problems. Experience of multiple life events was also significantly associated with externalizing problems, increasing the risk up to four times (Table 5).

**Table 5:** Multiple logistic regression predicting externalizing problems at age 3

	Odds ratio	95.0 % CI for OR	*p*-value
Externalizing ≥ 90th percentile	r^2^ = 0.09		
EPDS	2.595	1.390–4.844	0.003
Life events	4.024	1.994–8.119	<0.001
Parental unemployment	0.322	0.184–0.563	<0.001
Aggressive ≥ 90th percentile	r^2^ = 0.06		
Life events	3.515	1.695–7.289	0.001
Parental unemployment	0.358	0.199–0.647	0.001
Destructive ≥ 90th percentile	r^2^ = 0.12		
EPDS	2.794	1.493–5.228	0.001
Life events	2.474	1.137–5.383	0.022
Parental unemployment	0.318	0.180–0.561	<0.001
Sex	1.994	1.177–3.379	0.010
Apgar	8.535	1.920–37.947	0.005

*CI* confidence interval, *OR* odds ratio, *EPDS* Edinburgh postnatal depression scale, *CBCL* child behaviour checklist. Results concerning, Small for Gestational Age (SGA), smoking during pregnancy, birthweight, duration of gestation, neonatal illness, non-optimal pregnancy/birth, LSS, sex, *BDNF* and *5*-HTTLPR were not statistically significant and are not shown

Multiple Logistic regression. Dependent variables: CBCL (internalizing, withdrawn and anxious depressed). Independent variables (0 is used as a reference level): EPDS (0 ≤ 9, 1 ≥ 10), Life Events and LSS (0 < 90th percentile, 1 ≥ 90th percentile), Parental unemployment (0 = both parents employed, 1 = one or both parents unemployed), Parental immigration status (0 = both parents born in Sweden, 1 = one or both parents born abroad)

Parental unemployment was significantly associated with internalizing problems, as the risk of internalizing problems was doubled in children of whom one or both parents were unemployed (Table 4). Moreover, children of whom one parent or both parents were unemployed showed an increased risk of externalizing, aggressive and destructive problems, (Table 5).

No main effect was seen for either *5*-HTTLPR or *BDNF* Val66Met genotypes on behavioural problems in 3-year-old children. As noted above, life events predicted both internalizing and externalizing behavioural problems, but no gene-by-environment or gene-by-gene-by-environment interaction was found. Analyses were also run with genetic markers as dichotomous variables, comparing Met-carriers with Val/Val homozygotes and s-carriers with l/l homozygotes (data not shown). The results for internalizing scales remained the same, and for externalizing scales an interaction effect between *BDNF* and sex emerged. However, taking into consideration that numerous tests had been performed and that the statistical significance was only borderline (*p* = 0.044) it was decided not to replace the models. There was also an interaction effect between BDNF and parental immigration status for aggressive problems. However, the confidence interval spanned between 1.025 and 97.697, making interpretation of the result difficult.

A low Apgar score at birth predicted destructive problems, but with only this exception, pregnancy and birth-related factors were not significantly associated with either internalizing or externalizing problems. Boys showed an increased risk of destructive problems compared to girls, but no other sex differences were detected (Table 5).

## Discussion

This study examined the impact of early biological and social adversity on child behaviour at age 3. The longitudinal approach of the SESBiC study provides a unique opportunity to follow individuals from in-utero to early childhood. Multiple risk factors, genetic as well as environmental, have been included in the analysis to broaden the understanding of early predictors of child behavioural problems.

First, maternal symptoms of depression postpartum were found to be one of the most consistent and powerful predictors of behavioural problems at age 3, increasing the risk of internalizing as well as externalizing problems. Maternal depression has been shown to predict behavioural problems in children [45, 46], and postpartum depression has been found to affect child behaviour into adolescence [47].

The pathway from maternal depressive symptoms to child behavioural problems is probably multifactorial. Constitutional factors, unsatisfactory mother-child attachment and social consequences of depression are all likely to contribute. In this study the relationship between maternal depressive symptoms and child behaviour persisted when a wide range of risk factors were included in the model, emphasizing the importance of maternal mental health. Previously published results on the participants in this study showed no long term increase of behavioural problems in preadolescent children whose mothers reported symptoms of depression postpartum [27]. This could indicate that maternal mental health is most important in the present or in the short term, and that the opportunities for child recovery are good. It might also indicate that different factors influence the risk of behavioural problems at different ages and stages of development. The impact of maternal mental health on child behaviour shown in this study illustrates the need for knowledge and means to support women with symptoms of postpartum depression.

Secondly, the *5*-HTTLPR and *BDNF* Val66Met polymorphisms were not found to be predictive of behavioural problems at age 3 and no gene-by-environment or gene-by-gene-by-environment interaction was found. However, carriers of the Val/Met genotype of the *BDNF* Val66Met were at increased risk of anxious depressive problems compared to Val/Val carriers, although this effect disappeared in the multivariate analyses. The identification of an association between the Met allele and anxiety and depression is in line with previous results [11]. A main effect of the *5*-HTTLPR polymorphism on internalizing symptoms has been shown in this population at age 12 [27], thus not excluding main gene effects and calling for further analyses and follow-ups.

There is an inconsistency regarding gene-by-environment studies and depression, and methodological issues have been proposed to explain part of the discrepancy [11]. The character of adversities used might influence the results. In this study, life events comprised a broad spectrum of events including changing place of residence or starting day-care, while other studies focused more distinctively on childhood maltreatment [15]. At the age of 3, the number of life events experienced is, however, generally limited, although experience of multiple life events was shown to be a stable predictor of both internalizing and externalizing problems. To our knowledge no other study exploring the three-way interaction of *5*-HTTLPR, *BDNF* Val66Met and life events has focused on behavioural problems at an age as young as 3 years. Gene-by-environment studies assessing either *5-*HTTLPR or *BDNF* Val66Met separately or with different outcomes have been carried out on preschool children and infants [10, 48, 49]. However, different aims and methodologies make it difficult to draw generalizable conclusions about the impact of *5-*HTTLPR and *BDNF* Val66Met on behaviour in early childhood. Several studies have found gene-by-environment effects on depression and internalizing symptoms in adolescents and adults, and one reason for the non-significant results in this study could be the difference in the point in time at which problems were assessed. There is a request for well-powered, longitudinal gene-by-environment studies [19], and the design of the SESBiC study enables following the cohort into adulthood providing a good basis for future studies.

Thirdly, socio-environmental factors were shown to exert a major impact on child behaviour at the age of 3. Interestingly, parental immigration status mainly influenced internalizing problems, while parental unemployment was found to predict both internalizing and externalizing type of problems. There is evidence of an increased prevalence of behavioural problems in preschool aged children of immigrants [50], although most studies concern refugees and not children born in the host country. Second generation immigrants represent around 30 % of all newborns in Sweden, indicating that mental health and adjustment in this group should be a concern for society.

The fact that unemployment does imply negative consequences for families is generally accepted [51]. Unemployment could affect many aspects of family social and financial conditions, and studies have shown increased risk of behavioural problems in children experiencing socioeconomic hardship [52]. The findings in this study emphasize the importance of environmental factors for child wellbeing in preschool ages. Previous studies have argued for diminishing importance of socioeconomic conditions during adolescence [53], consequently these factors ought to be taken into consideration particularly when studying younger children.

Pregnancy and birth-related factors did not influence behavioural problems at age 3 to the extent expected. A low Apgar score did, however, increase the risk of destructive problems in the multivariate analysis, although groups were small and results must consequently be interpreted with caution. Perinatal risk factors have previously been studied mainly in relation to externalizing-type problems. Wagner et al. 2009 [3] found limited effect of obstetrical and neonatal complications on externalizing behavioural problems, although there is convincing support in the literature for behavioural problems in children born preterm [5]. This birth cohort initially included 88 % of newborns in the catchment area, and no data on the children whose mothers choose not to take part is available. It could be possible that mothers declined to take part due to early complications or psychosocial stress.

Given the previous findings of the SESBiC study, the results of the present study further emphasizes the importance of maternal mental health, especially concurrent or on short term. The present study also adds to the null findings of gene-by environment interactions of *5*-HTTLPR and *BDNF* Val66Met on internalizing and externalizing symptoms in preschool age. A recent meta-analysis on the gene-by environment interaction effects between *BDNF* Val66Met and stress on depression, lists two possible reasons for null findings: firstly the use of child and adolescent cohorts and secondly the use of objective measurements such as interviews [16]. According to this, the young age of the present study population could be the reason for the lack of gene-by-environment effects. With the background of this argument, the finding calls for replication studies specifically in children, and raises the question whether the three-way interaction effect varies across the life time.

This study has some limitations that must be pointed out. First, information on child behaviour was obtained from their mothers only. Previous studies have shown that maternal mood and wellbeing tend to have an impact on reports on child behaviour [54], however others have stated that the amount of variance explained by maternal depression is negligible and does not bias the results to a great extent [55]. Child behaviour should be assessed by a person knowing the child well, and by the age of 3 this would generally be the parents. Paternal factors other than unemployment were not included in this study. Paternal variables have been shown to influence child behaviour, although maternal factors might be of greater importance [56].

Although 84 % (*n* = 1,452) took part in the three year follow-up, only 65 % (*n* = 1,106) provided saliva samples at the 12 year follow-up, which limited the numbers in analysis to 1,106. Longitudinal multi-wave studies are often burdened with relatively large dropout rates, however in this case dropout analysis revealed differences between participants and non-participants. Parental immigration status was found to be an important predictor of internalizing problems so the comparably larger dropout rate among immigrant families is a problem. At the same time, a higher participation rate among children of immigrant parents would likely have had enhanced the results. Furthermore, no methods to adjust for missing data were utilized. This approach was chosen to achieve more accurate estimates using data on individuals that there were complete data for at both times measurement occurred. However, as mentioned above, missing data could possibly have reduced the variance on the outcomes.

## Conclusions

In summary, this study shows the importance of the psychosocial environment for psychosocial health in preschool children, illustrating the importance of maternal mental health and socio-environmental factors. No gene-by-environment interaction effect of *5*-HTTLPR and *BDNF* Val66Met was found at the age of 3, calling for more studies on this age group. The preconditions for early intervention via nationwide child welfare are good in Sweden, if identification of children at risk can be made. This study adds to existing literature concerning the factors that increase the risk of early behavioural problems and indicates areas to focus on for early identification of children at risk of dysfunctional development.

## Abbreviations

*5*-HTTLPR, serotonin transporter gene-linked polymorphic region; BDNF, Brain Derived Neurotropic Factor; CBCL, Child Behaviour Checklist; CI, Confidence Interval; CLES, Coddington Life Event Scale; EPDS, Edinburgh Postnatal Depression Scale; LSS, Life Stress Score; MBR, Medical Birth Register; OR, Odds Ratio; SESBiC study, South East Sweden Birth Cohort study; SGA, Small for Gestational Age; SNP, Single Nucleotide Polymorphism

## Additional file

Additional file 1: Table S1. Supplementary material. Correlations between genetic polymorphisms, birth characteristics, psychological scales and control variables (*n* = 1106). (DOC 73 kb)
