# Supplementary material for: Early predictors of behavioural problems in pre-schoolers – a longitudinal study of constitutional and environmental main and interaction effects
Source: BMC Pediatr. 2016 Jun 7;16:76. doi: 10.1186/s12887-016-0614-x (PMC4895962; doi:10.1186/s12887-016-0614-x)
Supplement: Supplementary file 1 — Supplementary material. Correlations between genetic polymorphisms, birth characteristics, psychological scales and control variables (n = 1106). (DOC 73 kb) [file 12887_2016_614_MOESM1_ESM.doc]

**Table S1.** Supplementary material**.** Correlations between genetic polymorphisms, birth characteristics, psychological scales and control variables (n = 1106).

|  | *BDNF*  Val66Met | *5*-HTTLPR | Apgar | Birthweight | Gestational length | SGA | Smoking | Neonatal  illness | Non-optimal  Birth | EPDS | Life Events | LSS | Parental  employment | Sex | Parental  immigration | CBCL  internalizing | CBCL a/d | CBCL wd | CBCL ext | CBCL ag | CBCL des |
| --- | --- | --- | --- | --- | --- | --- | --- | --- | --- | --- | --- | --- | --- | --- | --- | --- | --- | --- | --- | --- | --- |
| *BDNF* Val66Met | 1 |  |  |  |  |  |  |  |  |  |  |  |  |  |  |  |  |  |  |  |  |
| *5*-HTTLPR | .035 | 1 |  |  |  |  |  |  |  |  |  |  |  |  |  |  |  |  |  |  |  |
| Apgar 5 min | -.057 | -.015 | 1 |  |  |  |  |  |  |  |  |  |  |  |  |  |  |  |  |  |  |
| Birthweight low | -.035 | .031 | .033 | 1 |  |  |  |  |  |  |  |  |  |  |  |  |  |  |  |  |  |
| Gestational length preterm | 0.023 | .035 | -.023 | .499** | 1 |  |  |  |  |  |  |  |  |  |  |  |  |  |  |  |  |
| SGA | 0.12 | .008 | -.015 | .503** | .082** | 1 |  |  |  |  |  |  |  |  |  |  |  |  |  |  |  |
| Smoking during pregnancy | -.044 | .009 | .027 | .042 | .003 | .067* | 1 |  |  |  |  |  |  |  |  |  |  |  |  |  |  |
| Neonatal illness | .026 | -.012 | .091** | .227** | .348** | .057 | .026 | 1 |  |  |  |  |  |  |  |  |  |  |  |  |  |
| Non-optimal pregnancy/birth | -.044 | .012 | .177** | .296** | .368** | .244** | .725** | .524** | 1 |  |  |  |  |  |  |  |  |  |  |  |  |
| EPDS | .053 | -.051 | .019 | -.011 | -.007 | .015 | .017 | -.007 | .016 | 1 |  |  |  |  |  |  |  |  |  |  |  |
| Life Events | .054 | .000 | .012 | .009 | .003 | .095** | .085** | -.025 | .074* | .007 | 1 |  |  |  |  |  |  |  |  |  |  |
| LSS | -.020 | .003 | .107** | .075* | .024 | .095** | .181** | .075* | .188** | .172** | .081* | 1 |  |  |  |  |  |  |  |  |  |
| Parental employment | .054 | .020 | .029 | -.002 | -.036 | .024 | .057 | .014 | .025 | .060 | .056 | .134** | 1 |  |  |  |  |  |  |  |  |
| Sex | -.043 | .035 | -.016 | .061* | -.031 | 0.22 | -.008 | -.030 | -.017 | -.038 | .067* | .001 | .-.054 | 1 |  |  |  |  |  |  |  |
| Parental immigration status | .038 | .000 | .017 | -.030 | -.024 | -.010 | -.022 | -.011 | -.032 | .139** | .084** | .076* | .175** | -.088** | 1 |  |  |  |  |  |  |
| CBCL scales: |  |  |  |  |  |  |  |  |  |  |  |  |  |  |  |  |  |  |  |  |  |
| internalizing (CBCLint) | .001 | .018 | .037 | -.027 | -.014 | -0.11 | -.011 | -.009 | -.015 | .168** | .120** | .081* | .119** | .018 | .133** | 1 |  |  |  |  |  |
| anxious depressed (CBCLa/d) | .050 | .032 | .061 | -.011 | .057 | .005 | .022 | .034 | .045 | .110** | .127** | .102** | .093** | -.040 | .140** | .664** | 1 |  |  |  |  |
| withdrawn (CBCLwd) | -.007 | .027 | .015 | -.014 | -.013 | -.033 | .000 | -.019 | -.010 | .129** | .118** | .057 | .094** | 0.10 | .141** | .723** | .389** | 1 |  |  |  |
| externalizing (CBCLext) | .035 | .020 | .036 | .017 | .002 | .044 | .016 | .027 | .047 | .129** | .175** | .122** | .153** | -.054 | .071* | .505** | .396** | .404** | 1 |  |  |
| aggressive (CBCLag) | .022 | .028 | .006 | .000 | -.026 | .021 | 0.12 | .014 | .020 | .075* | .148** | .062 | .126** | -.005 | .013 | .478** | .343** | .388** | .757** | 1 |  |
| destructive (CBCL des) | .011 | -.008 | .106** | -.028 | -.032 | .045 | .028 | .028 | .066* | .131** | .103** | .124** | .155** | -.080* | .108** | .391** | .302** | .329** | .605** | .392** | 1 |

Note: CBCL = Child Behaviour Checklist for Children, EPDS = Edinburgh Postnatal Depresion Scale, LSS = Life Stress Score.

Pearson Correlation. Significance p < 0.05 indicated with *, p < 0.01 indicated with **.
